# Hospitalization outcome of heart diseases between patients who received medical care by cardiologists and non-cardiologist physicians: A propensity-score matched study

**DOI:** 10.1371/journal.pone.0235207

**Published:** 2020-07-06

**Authors:** Yu-Ming Wu, Chih-Chung Liu, Chun-Chieh Yeh, Li-Chin Sung, Chao-Shun Lin, Yih-Giun Cherng, Ta-Liang Chen, Chien-Chang Liao

**Affiliations:** 1 Department of Anesthesiology, Shuang Ho Hospital, Taipei Medical University, New Taipei City, Taiwan; 2 Department of Anesthesiology, School of Medicine, College of Medicine, Taipei Medical University, Taipei, Taiwan; 3 Department of Anesthesiology, Taipei Medical University Hospital, Taipei, Taiwan; 4 Anesthesiology and Health Policy Research Center, Taipei Medical University Hospital, Taipei, Taiwan; 5 Department of Surgery, China Medical University Hospital, Taichung, Taiwan; 6 Department of Surgery, University of Illinois, Chicago, IL, United States of America; 7 Division of Cardiology, Department of Internal Medicine, Shuang Ho Hospital, Taipei Medical University, New Taipei City, Taiwan; 8 Division of Cardiology, Department of Internal Medicine, School of Medicine, College of Medicine, Taipei Medical University, Taipei, Taiwan; 9 Taipei Heart Institute, Taipei Medical University, Taipei, Taiwan; 10 Department of Anesthesiology, Wan Fang Hospital, Taipei Medical University, Taipei, Taiwan; 11 Research Center of Big Data and Meta-Analysis, Wan Fang Hospital, Taipei Medical University, Taipei, Taiwan; 12 School of Chinese Medicine, College of Chinese Medicine, China Medical University, Taichung, Taiwan; University of Tennessee Health Science Center, UNITED STATES

## Abstract

**Background and aims:**

The effects of physician specialty on the outcome of heart disease remains incompletely understood because of inconsistent findings from some previous studies. Our purpose is to compare the admission outcomes of heart disease in patients receiving care by cardiologists and noncardiologist (NC) physicians.

**Methods:**

Using reimbursement claims data of Taiwan’s National Health Insurance from 2008–2013, we conducted a matched study of 6264 patients aged ≥20 years who received a cardiologist’s care during admission for heart disease. Using a propensity score matching procedure adjusted for sociodemographic characteristics, medical condition, and type of heart disease, 6264 controls who received an NC physician’s care were selected. Logistic regressions were used to calculate odds ratios (ORs) with 95% confidence intervals (CIs) for complications and mortality during admission for heart disease associated with a cardiologist’s care.

**Results:**

Patients who received a cardiologist’s care had a lower risk of pneumonia (OR = 0.61; 95% CI, 0.53–0.70), septicemia (OR = 0.49; 95% CI, 0.39–0.61), urinary tract infection (OR = 0.76; 95% CI, 0.66–0.88), and in-hospital mortality (OR = 0.37; 95% CI, 0.29–0.47) than did patients who received an NC physician’s care. The association between a cardiologist’s care and reduced adverse events following admission was significant in both sexes and in patients aged ≥40 years.

**Conclusion:**

We raised the possibility that cardiologist care was associated with reduced infectious complications and mortality among patients who were admitted due to heart disease.

## Introduction

Cardiovascular diseases, which include diseases of the heart and blood vessels caused by atherosclerosis, have been identified as one of the leading causes of death globally [1]. An estimated 17.7 million people died from cardiovascular diseases in 2015, representing 31% of all global deaths [2]. Additionally, cardiovascular diseases contribute extensively to the escalating costs of healthcare, which has become a worldwide burden. The annual direct cost of cardiovascular diseases was estimated at $250.8 billion in 2012–2013 in the United States and about €106 billion in Europe [3], representing approximately 9% of the total healthcare expenditure across the European Union in 2009 [4].

While caring for patients with cardiovascular diseases, cardiologists are more likely to use clinical guideline-supported therapies or be more knowledgeable about therapies than general physicians, a fact which has been shown to reduce mortality [5]. Previous studies reported that patients treated by cardiologists may have a lower mortality or better outcome than general physicians [6–13]. However, other studies reported that there was no significant difference in the patients’ outcomes between cardiologists and general physicians [14–17]. The inconsistent findings from previous studies may be due to the presence of some limitations, such as small sample sizes [10], the lack of matching procedures [6–10,14–16], inadequate control for confounding factors [7,8,11], and a focus on specific cardiovascular diseases or populations [6–11,14–16].

Limited information is available regarding the effects of a physician’s specialty on the outcomes of admission due to cardiovascular diseases in the Asian population. Using the claims data of national health insurance database, we conducted a population-based study to investigate the association between specialty care and admission outcomes in hospitalized patients suffering from cardiovascular diseases.

## Methods

### Source of data

The data used in this study were obtained from the reimbursement claims of Taiwan’s National Health Insurance, which contains inpatient and outpatient demographic characteristics, physicians’ primary and secondary diagnoses, treatment procedures, prescriptions, and medical expenditures. More than 99% of people in Taiwan received medical services from the National Health Insurance that cooperated with 471 hospitals and 20692 clinics in June 2018. Several scientific articles based on data from Taiwan’s National Health Insurance have been accepted in outstanding journals [18–20].

As these reimbursement claims were used in this study, the electronic database was decoded with patient identifications scrambled for further academic access for research to protect privacy. Although the National Health Research Institutes exempt such uses from informed consent, the guidelines of the Helsinki Declaration were obeyed during the execution of this study. This study was approved by the institutional review board of Taipei Medical University (TMU-JIRB-201701050; TMU-JIRB-201808012; TMU-JIRB-202006057).

### Study design

From the database of Taiwan’s National Health Insurance, we identified 34553 patients with a nonsurgical admission due to heart disease (HD) in 2008–2013, with 23482 of them receiving inpatient care by a cardiologist. To obtain the appropriate study subjects, we used a propensity-score matching technique to select 6264 patients receiving a cardiologist’s inpatient care and 6264 patients receiving inpatient care by a noncardiologist (NC) physician. Factors in the propensity-score matching included Sociodemographics (such as age, sex, low income), types of HD, history of disease (such as hypertension, diabetes, mental disorders, chronic obstructive pulmonary disease, hyperlipidemia, chronic kidney disease, end-stage renal disease, liver cirrhosis, and Parkinson’s disease), number of recent hospitalizations, and number of recent emergency visits. We compared the complications, mortality, intensity of care, length of hospital stay, medical expenditures during the admission due to HD between patients receiving inpatient care by a cardiologist and those receiving inpatient care by NC physicians.

### Measures and definition

The criterion of low income used in this study is based on the definition from the Bureau of National Health Insurance in Taiwan and details were described in our previous report [18–20]. Previous medical use before admission due to HD was considered as a potential covariate in this study along with number of emergency visits and hospitalizations. Based on the administration code and The International Classification of Diseases, Ninth Revision, Clinical Modification (ICD-9-CM), coexisting medical conditions were determined from medical claims for the 24-month preadmission period including hypertension (ICD-9-CM codes 401–405), diabetes (ICD-9-CM code 250), mental disorders (ICD-9-CM codes 290–319), chronic obstructive pulmonary disease (ICD-9-CM codes 491,492, 496), hyperlipidemia, chronic kidney disease, end-stage renal disease (D8 and D9), liver cirrhosis (ICD-9-CM codes 571.2, 571.5, 571.6), and Parkinson’s disease (ICD-9-CM code 332). Types of HD were also identified by the ICD-9-CM codes including myocardial infarction (ICD-9-CM codes 410, 412), other acute and subacute ischemic heart disease (ICD-9-CM code 411), angina pectoris (ICD-9-CM code 413), other chronic ischemic heart disease (ICD-9-CM code 414), acute and subacute endocarditis (ICD-9-CM code 421), acute myocarditis (ICD-9-CM code 422), other diseases of pericardium (ICD-9-CM code 423), other diseases of endocardium (ICD-9-CM code 424), cardiomyopathy (ICD-9-CM code 425), conduction disorders (ICD-9-CM code 426), cardiac dysrhythmias (ICD-9-CM code 427), heart failure (ICD-9-CM code 428), and ill-defined descriptions and complications of heart disease (ICD-9-CM code 429). Pneumonia (ICD-9-CM codes 480–486), septicemia (ICD-9-CM codes 038 and 998.5), urinary tract infection (ICD-9-CM code 599.0), mortality, length of hospital stay, and medical expenditures during the patients’ stays were considered as study outcomes. In this study, the non-cardiologist physicians included physicians with medical specialists in general medicine, family medicine, neurology, nephrology, gastroenterology, thoracic medicine, endocrinology, and infectious disease.

### Statistical analysis

The propensity score-matched pair analysis was used to examine the associations between physician specialty and outcomes of HD admission. A nonparsimonious multivariable logistic regression model was used to estimate a propensity score for patients receiving a cardiologist’s service or not. Covariates in this model included age, sex, low income, types of HD, hypertension, diabetes, mental disorders, chronic obstructive pulmonary disease, hyperlipidemia, chronic kidney disease, end-stage renal disease, liver cirrhosis, Parkinson’s disease, number of hospitalizations, and number of emergency visits. We matched the cardiologists’ patients to the NC physicians’ patients, using a greedy matching algorithm (without replacement) with a caliper width of 0.2 SDs of the log odds of the estimated propensity score. Categorical variables between cardiologists’ patients and NC physicians’ patients were analyzed by using frequencies (percentages) and chi-square tests. Continuous variables between cardiologists’ patients and NC physicians’ patients are presented as means ± standard deviations and analyzed using t tests. We used logistic regression to calculate the adjusted odds ratios (ORs) and 95% confidence intervals (CIs) of the outcomes of HD admission associated with physician specialty. In addition, subgroup analysis was also used to stratify the subjects according to age, sex, number of medical conditions, and type of HD, to examine the outcomes of HD admission between patients receiving a cardiologist’s care or not in these strata.

## Results

Table 1 shows the baseline characteristics of the patients suffering from cardiovascular diseases and the controls who underwent different specialty physician cares. After the propensity score matching, there were no significant differences in the groups including the patients suffering from cardiovascular diseases with and without a cardiologist’s care analyzed by age, sex, low income, medical conditions (hypertension, diabetes, mental disorders, chronic obstructive pulmonary disease, hyperlipidemia, chronic kidney disease, end-stage renal disease, liver cirrhosis and Parkinson’s disease), type of HD, number of hospitalizations and number of emergency visits.

**Table 1:** Characteristics of patients with cardiovascular admission receiving care by cardiologists and NC physicians.

	NC physicians (N = 6264)		Cardiologists (N = 6264)		*P* value
Sex	n	(%)	n	(%)	1.0000
Female	2519	(40.2)	2519	(40.2)	
Male	3745	(59.8)	3745	(59.8)	
Age, years					1.0000
20–29	70	(1.1)	70	(1.1)	
30–39	169	(2.7)	169	(2.7)	
40–49	503	(8.0)	503	(8.0)	
50–59	1099	(17.5)	1099	(17.5)	
60–69	1270	(20.3)	1270	(20.3)	
70–79	1825	(29.1)	1825	(29.1)	
≥80	1328	(21.2)	1328	(21.2)	
Low income					1.0000
No	6160	(98.3)	6160	(98.3)	
Yes	104	(1.7)	104	(1.7)	
Medical conditions					
Hypertension	3044	(48.6)	3044	(48.6)	1.0000
Diabetes	1218	(19.4)	1218	(19.4)	1.0000
Mental disorders	816	(13.0)	816	(13.0)	1.0000
COPD	713	(11.4)	713	(11.4)	1.0000
Hyperlipidemia	235	(3.8)	235	(3.8)	1.0000
Chronic kidney disease	95	(1.5)	95	(1.5)	1.0000
End-stage renal disease	47	(0.8)	47	(0.8)	1.0000
Liver cirrhosis	32	(0.5)	32	(0.5)	1.0000
Parkinson’s disease	24	(0.4)	24	(0.4)	1.0000
Type of heart disease					1.0000
Acute myocardial infarction	1014	(16.2)	1014	(16.2)	
Other acute and subacute IHD	254	(4.1)	254	(4.1)	
Angina pectoris	286	(4.6)	286	(4.6)	
Other chronic IHD	2199	(35.1)	2199	(35.1)	
Acute and subacute endocarditis	25	(0.4)	25	(0.4)	
Acute myocarditis	4	(0.1)	4	(0.1)	
Other diseases of pericardium	11	(0.2)	11	(0.2)	
Other diseases of endocardium	89	(1.4)	89	(1.4)	
Cardiomyopathy	43	(0.7)	43	(0.7)	
Conduction disorders	57	(0.9)	57	(0.9)	
Cardiac dysrhythmias	892	(14.2)	892	(14.2)	
Heart failure	1383	(22.1)	1383	(22.1)	
Ill-defined descriptions and complications of heart disease	7	(0.1)	7	(0.1)	
Number of hospitalizations					1.0000
0	4173	(66.6)	4173	(66.6)	
1	1184	(18.9)	1184	(18.9)	
2	352	(5.6)	352	(5.6)	
≥3	555	(8.9)	555	(8.9)	
Number of emergency visits					1.0000
0	2956	(47.2)	2956	(47.2)	
1	1547	(24.7)	1547	(24.7)	
2	703	(11.2)	703	(11.2)	
≥3	1058	(16.9)	1058	(16.9)	

COPD, chronic obstructive pulmonary disease; NC, non-cardiologist; IHD, ischemic heart disease.

Compared with HD patients receiving medical service by an NC physician (Table 2), those receiving care by a cardiologist had lower risks of pneumonia (OR = 0.61; 95% CI, 0.53–0.70), septicemia (OR = 0.49; 95% CI, 0.39–0.61), urinary tract infection (OR = 0.76; 95% CI, 0.66–0.88), and in-hospital mortality (OR = 0.37; 95% CI, 0.29–0.47). The average length of hospital stay (7.7±19.7 vs 5.7±7.0 days; *P* < .001) was shorter in cardiologists’ patients than in NC physicians’ patients.

**Table 2:** Adverse events after admission of heart disease in patients who received care by cardiologists and NC physicians.

	NC physicians (N = 6264)		Cardiologists (N = 6264)		Outcome risk	
Outcomes after admission	Events	%	Event	%	OR	(95% CI)^b^
30-day in-hospital mortality	249	4.0	94	1.5	0.37	(0.29–0.47)
Pneumonia	530	8.5	334	5.3	0.61	(0.53–0.70)
Septicemia	238	3.8	118	1.9	0.49	(0.39–0.61)
Urinary tract infection	429	6.9	331	5.3	0.76	(0.66–0.88)
ICU stay	2072	33.1	2139	34.2	1.05	(0.97–1.13)
Medical expenditure, USD^c^	2454±3053		2542±2645		p = 0.0860	
Length of hospital stay, days^c^	7.7±19.7		5.7±7.0		p<0.0001	

NC, non-cardiologist; CI, confidence interval; OR, odds ratio.

^b^Analyzed by univariate logistic regressions after propensity-score matching.

^c^Mean±SD; In the multiple regressions, the medical expenditure (beta = 87.6, *P* = .0774) and length of hospital stay (beta = -1.98, *P* < .0001) were associated with cardiologists.

The stratified analysis show that the association between cardiology specialty and reduced postadmission adverse events was significant in females (OR = 0.66; 95% CI, 0.57–0.76), males (OR = 0.59; 95% CI, 0.51–0.67), and people aged 40–49 years (OR = 0.55; 95% CI, 0.33–0.92), 50–59 years (OR = 0.56; 95% CI, 0.41–0.79), 60–69 years (OR = 0.58; 95% CI, 0.45–0.76), 70–79 years (OR = 0.64; 95% CI, 0.54–0.76), and ≥80 years (OR = 0.60; 95% CI, 0.51–0.72) (Table 3).

**Table 3:** The stratified analysis for the adverse events after admission of heart disease associated with cardiologists’ care.

		Adverse events^a^				
		n	Events	Rate, %	OR	(95% CI)^b^
Female	NC physicians	2519	580	23.0	1.00	(reference)
	Cardiologists	2519	414	16.4	0.66	(0.57–0.76)
Male	NC physicians	3745	574	15.3	1.00	(reference)
	Cardiologists	3745	359	9.6	0.59	(0.51–0.67)
Age 20–39 years	NC physicians	239	26	10.9	1.00	(reference)
	Cardiologists	239	17	7.1	0.63	(0.33–1.19)
Age 40–49 years	NC physicians	503	42	8.4	1.00	(reference)
	Cardiologists	503	24	4.8	0.55	(0.33–0.92)
Age 50–59 years	NC physicians	1099	102	9.3	1.00	(reference)
	Cardiologists	1099	60	5.5	0.56	(0.41–0.79)
Age 60–69 years	NC physicians	1270	159	12.5	1.00	(reference)
	Cardiologists	1270	98	7.7	0.58	(0.45–0.76)
Age 70–79 years	NC physicians	1825	385	21.1	1.00	(reference)
	Cardiologists	1825	268	14.7	0.64	(0.54–0.76)
Age ≥80 years	NC physicians	1328	440	33.1	1.00	(reference)
	Cardiologists	1328	306	23.0	0.60	(0.51–0.72)
0 medical condition	NC physicians	2051	362	17.7	1.00	(reference)
	Cardiologists	2051	222	10.8	0.57	(0.47–0.68)
1 medical condition	NC physicians	2586	472	18.3	1.00	(reference)
	Cardiologists	2586	326	12.6	0.65	(0.55–0.75)
≥2 medical conditions	NC physicians	1627	320	19.7	1.00	(reference)
	Cardiologists	1627	225	13.8	0.66	(0.54–0.79)
0 hospitalizations	NC physicians	4173	649	15.6	1.00	(reference)
	Cardiologists	4173	436	10.5	0.63	(0.56–0.72)
1 hospitalizations	NC physicians	1184	240	20.3	1.00	(reference)
	Cardiologists	1184	159	13.4	0.61	(0.49–0.76)
2 hospitalizations	NC physicians	352	90	25.6	1.00	(reference)
	Cardiologists	352	62	17.6	0.62	(0.43–0.90)
≥3 hospitalizations	NC physicians	555	175	31.5	1.00	(reference)
	Cardiologists	555	116	20.9	0.57	(0.44–0.75)
0 emergency visits	NC physicians	2956	473	16.0	1.00	(reference)
	Cardiologists	2956	318	10.8	0.63	(0.54–0.74)
1 emergency visits	NC physicians	1547	307	19.8	1.00	(reference)
	Cardiologists	1547	192	12.4	0.57	(0.47–0.70)
2 emergency visits	NC physicians	703	141	20.1	1.00	(reference)
	Cardiologists	703	80	11.4	0.51	(0.38–0.69)
≥3 emergency visits	NC physicians	1058	233	22.0	1.00	(reference)
	Cardiologists	1058	183	17.3	0.74	(0.60–0.92)
No other chronic IHD	NC physicians	4065	993	24.4	1.00	(reference)
	Cardiologists	4065	648	15.9	0.59	(0.53–0.66)
Other chronic IHD	NC physicians	2199	161	7.3	1.00	(reference)
	Cardiologists	2199	125	5.7	0.76	(0.60–0.97)
No heart failure	NC physicians	4881	744	15.2	1.00	(reference)
	Cardiologists	4881	472	9.7	0.60	(0.53–0.67)
Heart failure	NC physicians	1383	410	29.7	1.00	(reference)
	Cardiologists	1383	301	21.8	0.66	(0.56–0.78)
No AMI	NC physicians	5250	886	16.9	1.00	(reference)
	Cardiologists	5250	592	11.3	0.63	(0.56–0.70)
AMI	NC physicians	1014	268	26.4	1.00	(reference)
	Cardiologists	1014	181	17.9	0.61	(0.49–0.75)
No cardiac dysrhythmias	NC physicians	5372	931	17.3	1.00	(reference)
	Cardiologists	5372	679	12.6	0.69	(0.62–0.77)
Cardiac dysrhythmias	NC physicians	892	223	25.0	1.00	(reference)
	Cardiologists	892	94	10.5	0.35	(0.27–0.46)

CI, confidence interval; NC, non-cardiologist; OR, odds ratio.

^a^Adverse events included with 30-day in-hospital mortality, pneumonia, septicemia, urinary tract infection.

^b^Analyzed by univariate logistic regressions after propensity-score matching.

## Discussion

In this population-based study with a propensity-score matching analysis, we found that patients admitted due to cardiovascular diseases receiving treatment by cardiologists had lower risks of pneumonia, septicemia and urinary tract infection and 30-day in-hospital mortality compared with those cared by general physicians. Shorter length of hospital stays was also noted in inpatients receiving a cardiologist’s medical services. The association between a cardiologist’s care and less adverse events following admission was significant in several of the subgroups including gender, age group of more than 40 years and patients with various medical conditions, previous emergency and inpatient care.

We proposed some possible explanations to clarify the beneficial effects of receiving a cardiologist’s care on the outcome of admission due to cardiovascular disease. First, the cardiologists tended to use more exact diagnostic procedures and were characterized by better adherence to certain evidence-based processes of care [8,9,12,14,21–24], such as beta-blockers [9,10,25–29], calcium channel blockers or angiotensin-converting enzyme inhibitors [28,29]. Second, a cardiologist is also associated with a better disease guideline compliance, which not only includes the acute phase of the disease medical care but also the disease risk factor survey, emergency care, management during hospitalization and at discharge, long-term therapies and complication management [12,13,15,30]. Third, the cardiologist is also more aggressive when selecting the most appropriate intervention procedure or when transferring the patient to a surgeon for operation if they are unable to cure the patient [8,14,18]. Although the previous study suggested that the differences of outcome disappeared after adjusting for differences in patient demographics and comorbidities between cardiologist and non-cardiologist treated patients [31], our study found the less infectious complications and mortality in patients received inpatient care by cardiologists after the propensity-score matching. It revealed that future well-design studies even randomized clinical trials are necessary for presenting the reliable evidence.

Fourth, another explanation is the distribution of cardiologists between hospitals and the complexity of the hospital. In Taiwan, the medical center usually has an integrated specialist departmental system, more medical resources and more cardiologists. A patient suffering from a cardiovascular disease admitted to the medical center may receive cardiologist’s care with more ease than others who are admitted to metropolitan hospitals. The relationship between cardiovascular mortality and physician volume, and hospital volume/complexity has been proved [10,32]. In our study database, which involves a national cohort, the type of hospital the patients were admitted to was not included. The difference in distribution between cardiologists and general physicians between hospitals may contribute to the disease outcome. In addition, the type of ward is also related to a patient’s health outcome. Patients admitted to the cardiovascular units at medical centers may benefit from more comprehensive care and a higher number of therapies [11,33–37]. Patients also receive better nursing care focusing on cardiovascular complications, better medical attention by doctors and stay in a less complex medical care unit. A simpler ward environment and patient population may also relate to a lower hospital acquired infection rate. Finally, an increased patient compliance with the attending physician’s instructions, due to a better doctor-patient relationship, is also a possible explanation. Past studies revealed that increasing adherence to attendance may decrease short-term readmission rate or mortality [12,38–40]. We observed that in Taiwan, it is at the discretion of the emergency physicians to shunt patients during admission in many regional or district hospitals. Emergency physicians may also interfere with assignment of the patients to their attending physician. For example, emergency physicians tend to increasingly assign new cases to general physicians, which may increase the misclassification rate.

We noticed that patients aged between 30–39 years old did not have better outcomes under a cardiologist’s care. The possible explanation for this is that patients in this age group are healthier and more resistant to diseases or have a more rapid rehabilitation ability. On the other hand, from the epidemiological point of view, this comprehensive outcome difference between general physicians and cardiologists definitely demonstrates that the attending physician’s specialty affects the quality of the cardiovascular disease treatment.

Interpretation of our findings should be done with caution because of some study limitations. First, information regarding the location of residence, lifestyle, family history of cardiovascular disease, clinical risk scores, clinical blood sample lab data and seniority of the doctors were unavailable in the database and these factors may be residual confounders in our study. The location of residence may be one of factors associated with patients to be in cardiologist group or NC group because the small hospital located in very rural area has no cardiology specialty. Second, we do not have detailed data regarding the consultation of a cardiologist during the inpatient care by the general physicians. However, this limitation may contribute to the underestimation of beneficial effects of a cardiologist’s services on inpatients with cardiovascular diseases. Third, we did not match the hospital complexity/volume in both groups, which may be a confounding factor as mentioned before and in past studies [10,32], because there are no related data in Taiwan’s National Health Insurance database. Type of hospital is one of the potential confounding factors in this study. Fourth, we also did not normalize the use of medications, diagnosis procedures and treatment between both groups, which were usually compared in earlier references as the major results and causes. In contrast with other studies that were focused on only one cardiovascular disease [6,8–11,14–15], our analysis included nearly all common cardiovascular diseases along with many subtypes. In addition, our study focused on short-term complications and mortality following admission due to cardiovascular diseases, excluding out-patient outcomes. In contrast, with previous reports [6,7,10,11,15,16], we believed that the short-term and admission outcomes may related to the type of physician service, thus underscoring the a cardiologist’s value. Finally, the validation of Taiwan’s National Health Insurance Research Database remains inadequate although the physician’s diagnosis and codes of diseases were validated in previous studies [41–46]. We also could not exclude the possibility of residual confounding variables although several potential confounders were adjusted for in our analysis models.

In conclusion, we raised that possibility that cardiologist care was associated with reduced infectious complications, mortality, and length of hospital stay among patients admitted due to HD. Our study implicates that the role of the physician specialty is crucial in the inpatient care of cardiovascular disease. Consultation with a cardiologist is encouraged when a general physician is providing medical care to patients with cardiovascular diseases.

## Supporting information

S1 Table Characteristics of patients with cardiovascular admission receiving care by cardiologists and NC physicians. (DOC)

## List of abbreviation

ICD-9-CM: International Code of Diseases, Ninth Edition, Clinical Modification
OR: odds ratio
CI: confidence interval
HD: heart disease
NC: noncardiologist
