# Supplementary material for: Hospitalization outcome of heart diseases between patients who received medical care by cardiologists and non-cardiologist physicians: A propensity-score matched study
Source: PLoS One. 2020 Jul 6;15(7):e0235207. doi: 10.1371/journal.pone.0235207 (PMC7338078; doi:10.1371/journal.pone.0235207)
Supplement: S1 Table — (DOC) [file pone.0235207.s001.doc]

| **S1 Table. Characteristics of patients with cardiovascular admission receiving care by cardiologists and NC physicians** | | | | | |
| --- | --- | --- | --- | --- | --- |
|  | NC physicians  (N=11071) | | Cardiologists  (N=23482) | | *P* value |
| Sex | n | (%) | n | (%) | 0.0001 |
| Female | 4748 | (42.9) | 9559 | (40.7) |  |
| Male | 6323 | (57.1) | 13923 | (59.3) |  |
| Age, years |  |  |  |  | <0.0001 |
| 20-29 | 173 | (1.6) | 343 | (1.5) |  |
| 30-39 | 345 | (3.1) | 785 | (3.3) |  |
| 40-49 | 859 | (7.8) | 2345 | (10.0) |  |
| 50-59 | 1705 | (15.4) | 4480 | (19.1) |  |
| 60-69 | 2122 | (19.2) | 5659 | (24.1) |  |
| 70-79 | 3177 | (28.7) | 6270 | (26.7) |  |
| ≥80 | 2690 | (24.3) | 3600 | (15.3) |  |
| Low income |  |  |  |  | <0.0001 |
| No | 10261 | (92.7) | 22380 | (95.3) |  |
| Yes | 810 | (7.3) | 1102 | (4.7) |  |
| Medical conditions |  |  |  |  |  |
| Hypertension | 5418 | (48.9) | 11398 | (48.5) | 0.4883 |
| Diabetes | 2817 | (25.4) | 5586 | (23.8) | 0.0008 |
| Mental disorders | 2303 | (20.8) | 4472 | (19.0) | 0.0001 |
| Chronic obstructive pulmonary disease | 2035 | (18.4) | 3120 | (13.3) | <0.0001 |
| Hyperlipidemia | 725 | (6.6) | 1740 | (7.4) | 0.0037 |
| Chronic kidney disease | 1015 | (9.2) | 1176 | (5.0) | <0.0001 |
| End-stage renal disease | 497 | (4.5) | 612 | (2.6) | <0.0001 |
| Liver cirrhosis | 311 | (2.8) | 626 | (2.7) | 0.4442 |
| Parkinson’s disease | 377 | (3.4) | 439 | (1.9) | <0.0001 |
| Type of heart disease |  |  |  |  | <0.0001 |
| Acute myocardial infarction | 1537 | (13.9) | 3623 | (15.4) |  |
| Other acute and subacute IHD | 592 | (5.4) | 1386 | (5.9) |  |
| Angina pectoris | 649 | (5.9) | 1408 | (6.0) |  |
| Other chronic IHD | 3004 | (27.1) | 8953 | (38.1) |  |
| Acute and subacute endocarditis | 179 | (1.6) | 77 | (0.3) |  |
| Acute myocarditis | 25 | (0.2) | 20 | (0.1) |  |
| Other diseases of pericardium | 90 | (0.8) | 75 | (0.3) |  |
| Other diseases of endocardium | 235 | (2.1) | 512 | (2.2) |  |
| Cardiomyopathy | 110 | (1.0) | 321 | (1.4) |  |
| Conduction disorders | 152 | (1.4) | 596 | (2.5) |  |
| Cardiac dysrhythmias | 1662 | (15.0) | 3276 | (14.0) |  |
| Heart failure | 2741 | (24.8) | 3160 | (13.5) |  |
| Ill-defined descriptions and  complications of heart disease | 95 | (0.8) | 75 | (0.3) |  |
| Number of hospitalizations |  |  |  |  | <0.0001 |
| 0 | 5496 | (49.6) | 14520 | (61.8) |  |
| 1 | 2346 | (21.2) | 4902 | (20.9) |  |
| 2 | 1157 | (10.5) | 1977 | (8.4) |  |
| ≥3 | 2072 | (18.7) | 2083 | (8.9) |  |
| Number of emergency visits |  |  |  |  | <0.0001 |
| 0 | 4203 | (38.0) | 10047 | (42.8) |  |
| 1 | 2615 | (23.6) | 5893 | (25.1) |  |
| 2 | 1565 | (14.1) | 3107 | (13.2) |  |
| ≥3 | 2688 | (24.3) | 4435 | (18.9) |  |
| NC, non-cardiologist; IHD, ischemic heart disease | | | | | |
